# Mulberry leaf polyphenols alleviated high-fat diet-induced obesity in mice

**DOI:** 10.3389/fnut.2022.979058

**Published:** 2022-09-15

**Authors:** Rui Li, Qubo Zhu, Xiaoyan Wang, Haiyan Wang

**Affiliations:** ^1^Chongqing Academy of Animal Sciences, Chongqing, China; ^2^Southwest University, Chongqing, China; ^3^National Center of Technology Innovation for Pigs, Chongqing, China

**Keywords:** mulberry leaf polyphenols, mice, obesity, fatty acid composition, adipose tissue browning

## Abstract

Mulberry leaf is an important medicinal food plant, which is rich in polyphenol compounds. Mulberry leaf polyphenols (MLP) possess significant lipid-lowering and antioxidant effects, and healthcare functions. In this study, the polyphenol content of mulberry leaf ethanol extract was measured using HPLC. The analysis of mulberry leaf extract resulted in the identification of 14 compounds, of which Chlorogenic acid and Quercitrin were the highest. A high-fat diet (HFD)-induced obese mouse model was developed and treated with MLP for 12 weeks to explore their effect on lipid metabolism in HFD-induced obese mice. The results showed that the MLP could inhibit the weight gain and fat cell volume increase in the HFD-induced obese mice in a dose-dependent manner. Further analysis revealed that the MLP decelerated the fatty acid composition in the adipose tissues of HFD-induced obese mice, and significantly increased the polyunsaturated-to-saturated fatty acid (PUFA/SFA) ratio. The real-time quantitative PCR (RT-qPCR) results indicated that the MLP significantly inhibited the down regulation of uncoupling protein (UCP) 1 (UCP1), UCP3, and PR domain zinc finger protein 16 (PRDM16) caused by the HFD. These beneficial effects of MLP on HFD-induced obese mice might be attributed to their ability to change the fatty acid composition of adipose tissue and increase the expression of thermogenesis genes. Overall, the study results suggested that the MLP could serve as potential lipid-lowering and weight-loss functional food and healthcare products.

## Introduction

Mulberry leaf, the dried leaf of *Morus alba* L., is widely acknowledged as a traditional Chinese herbal medicine (1). Mulberry leaf is rich in vitamins, minerals, and active ingredients, such as alkaloids, flavonoids, polysaccharides, and polyphenols, with the potential benefits of lowering blood sugar and blood lipids and antiaging, anticancer, and antibacterial effects (2, 3). Of the active ingredients, polyphenols are the fundamental compounds in mulberry leaves, contributing to their antioxidant effects (4).

Obesity has emerged as a global pandemic, eroding human health; thus, making it a major public health concern (5). Obesity is a chronic metabolic disease that disrupts physical health, such as the normal functions of the digestive and endocrine systems, and is often associated with various diseases, such as cardiovascular diseases, dyslipidemia, hyperlipidemia, and hypertension (6, 7). Obesity is mostly caused by high-calorie food intake, lack of physical exercise, and incorrect eating habits. Although the most effective approach to reducing obesity is to control diet and increase exercise (8), the application of weight loss drugs is the most favored method of weight loss for obese people (9). Therefore, finding effective drugs without apparent side effects, especially from natural products, has become the area of research interest in the field of weight loss (10).

Numerous studies have revealed that plant polyphenols may contribute to the prevention of obesity and metabolic syndrome. Chlorogenic acid and caffeine combination regulated the mRNA and protein levels of lipid metabolism-related genes in mice liver, and then inhibited fat synthesis and promoted lipid oxidation to achieve a slimming effect (11). Rutin improves the impaired insulin tolerance in SAMP8 mice fed a high-fat diet (HFD) (12). Quercetin and resveratrol in plant polyphenols are good SIRT1 agonists that can enhance PGC-1α and promote oxidative metabolism and mitochondrial genesis (13).

Mulberry leaf extract contains chlorogenic acid and its isomers, rutin, quercetin, resveratrol, quercetin, p-hydroxycinnamic acid, and caffeic acid (14). The role of MLP in anti-obesity has received increasing attention. A previous study reported that mulberry leaf ethanol extract decreases weight by acting as a melanin-concentrating hormone-1 antagonist in diet-induced obese mice (15). Mulberry leaf ethanol extract inhibited adipogenesis and the expression of adipogenesis-related factors in 3T3-L1 adipocytes (16). Therefore, MLP could be considered as potential weight loss and healthcare drugs. The anti-obesity mechanisms of MLP are multifaceted, which are not completely clear at present, and need to be further studied.

The mulberry leaf is also a high-quality domestic animal feed (17). Fatty acid composition is one of the effective indicators of the nutritional value of food (18). Will there be changes in the composition of the tissues of animals eating MLP? Is this change beneficial to the health of humans who use these animal products as food? With these questions in mind, the present study sought to explore the effects of MLP on HFD-induced obese mice and to analyze their potential value in improving animal products by evaluating their effects on fatty acid composition.

## Materials and methods

### Instrumentation and materials

The instruments used were AGILENGT 1260 High-efficiency liquid chromatography (Agilent Technologies, CA, United States), GC-MS 7890B-5977A (Agilent Technologies, CA, United States), and Milli-Q ultra-pure water purification system (Millipore, MA, United States). Gallic acid, gentisic acid, chlorogenic acid, vanillic acid, caffeic acid, syringic acid, epicatechin, rutin, hyperoside, benzoic acid, quercitrin, quercetin, kaempferol, and resveratrol were purchased from Beijing Solabao Technology Co., Ltd. (Beijing, China); with puritygreater than 98%. Quality spectrometer pure methanol (swedish Oceanpak, GOT, Sweden), mass spectrometry pure acetamin and methampite (Fisher, MFL, United States), and other reagents were all of analytical grade.

### Mulberry leaf polyphenol extraction

Approximately 1 kg of mulberry leaf powder was weighed and mixed with 70% ethanol according to the material concentration of 0.04 g/ml. The solution was subjected to ultrasonic extract for 60 min at 400 W and filtered with suction. The filtrate was concentrated under reduced pressure at 55°C and stored at 4°C. MLP samples were diluted 10 times in pure methanol before high-performance liquid chromatography (HPLC) analysis.

### High-performance liquid chromatography conditions

The mulberry polyphenol appraisal and detection methods used by Shen et al. (19) were modified. HPLC analysis was performed on an Alltima C18 column (4.6 mm × 250 mm, 5 μm). The flow phase A was acetylene and B was 0.2% acetic acid solution. The elution procedures were 0–10 min, 5% A; 10–40 min, 5–25% A; 40–45 min, 25–35% A; and 45–50 min, 35–50% A. The flow velocity was 1 ml/min, the running time was 55 min, the column temperature was 30°C, the amount of inlet was 5 μl, and the detection wavelength was 280 nm.

### Ethics statement

A total of 40 C57BL/6 healthy male mice (23.95 ± 0.81) g were procured from the Hunan SJA Laboratory Animal Co., Ltd., Chengdu, China. All the procedures and animal care were carried out in accordance with the requirements of the British Animal (Scientific Procedures) Act 1986. The experimental protocol was approved by the Animal Care and Ethics Committee of Chongqing Academy of Animal Sciences (No. cqaa2020007).

### Animals and treatment

All the experimental animals were housed in a dedicated animal room at (22 ± 3)°C with a 12-h light/12-h dark cycle and allowed to eat and drink freely. After 1 week of adaptation, the experimental animals were randomly divided into 4 groups, with 10 animals in each group. The NC group was fed with a normal diet; the HFD group was fed with special feed containing 40% fat; the HFD-H group was fed with special feed containing 40% fat and gavage of 200 μl of highly concentrated mulberry leaf polyphenol extract (the liquid from 1 kg mulberry leaves was concentrated after suction filtration and diluted in 500 ml water); the HFD-L group was fed with special feed containing 40% fat and gavage of 200 μl of low concentrated mulberry leaf polyphenol extract (half of the high concentration mulberry leaf extract). Gavage was administered at 10 a.m. every day, and normal saline was administered to the NC and HFD groups only.

### Organ index and Lee’s index


$$\mathrm{Organ}\mathrm{index}=\frac{\mathrm{organ}\mathrm{weight}}{\mathrm{body}\mathrm{weight}}\times 100\%$$

Lees′index=bodyweight×1000bodylength3

The unit of organ weight and body weight is g, and the unit of body length is cm.

### Blood test and tissue sectioning

The concentration of total cholesterol (TC) and triglyceride (TG) in the serum was determined using a kit (Nanjing Jiancheng Bioengineering Institute, Nanjing, China). The fasting blood glucose concentrations were measured using a blood glucose meter (Accu-Chek Active, Roche, Ireland) (20). After sectioning, the adipose tissue was fixed with 4% paraformaldehyde, dehydrated by ethanol, infiltrated and embedded in paraffin, and stained with hematoxylin and eosin (H&E) to analyze the cell size.

### Fatty acid composition analysis

The fatty acid composition test was performed according to the previously reported method (21). Gas chromatography-mass spectrometry (GC-MS 7890B-5977A, Agilent, United States) was used to detect the fatty acid composition. Then, 100 mg of adipose tissue was homogenized, 2 ml of *n*-hexane added, and it was shaken for 30 min at 50°C. Next, 3 ml of methanol solution (0.4 moL/L) was added and then shaken for 30 min at 50°C. Lastly, 1 ml of water was mixed with 2 ml of *n*-hexane and shaken for 20 min at (22 ± 3)°C. Afterward, the solution was allowed to stand for stratification, and the upper layer was separated for gas injection detection. Chromatographic column: DB-23 (30 m × 320 μm × 0.25 μm), the carrier gas was helium, inlet temperature was 250°C; the split ratio was 1/5; injection volume was 1 μl, detector temperature was 230°C, the column oven temperature was 50°C for 1 min, 25°C/min to 175°C, 4°C/min to 230°C for 24.75 min.

### Real-time quantitative polymerase chain reaction

Total RNA was extracted from mouse fat using TRIzol reagent (Invitrogen, Guangzhou, China). After reverse transcription, the mRNA level was detected using an RT-qPCR SYBR premixed Dalian kit (TaKaRa, Dalian, China) and a real-time PCR detection system (Bio-Rad, Richmond, CA, United States). ACTB was used as the mRNA internal control. The primer sequences are listed in Supplementary Table 1.

### Statistical analysis

All the data were expressed as mean ± standard deviation (SD). The analysis of variance (ANOVA) and significance of differences among the means were tested using a one-way ANOVA test and SPSS 20.0 software (SPSS Inc., NY, United States). *P* ≤ 0.05 was considered significant.

## Results

### Analysis of polyphenol monomer compounds in mulberry leaf polyphenols

The HPLC method was used to qualify and quantitatively analyze MLP. The following compounds were identified in mulberry leaf extract: dragon gallic acid, gentisic acid, chlorogenic acid, vanillic acid, caffeic acid, syringic acid, epicatechin, rutin, hyperoside, benzoic acid, quercitrin, quercetin, kaempferol, and resveratrol (14) polyphenol monomer compounds (Table 1).

**TABLE 1:** The polyphenol content of mulberry leaf polyphenols (MLP).

No.	Polyphenol monomer compounds	MLP mg/ml
1	Gallic acid	0.87 ± 0.04
2	Gentisic acid	1.73 ± 0.16
3	Chlorogenic acid	10.73 ± 0.52
4	Vanillic acid	2.24 ± 0.13
5	Caffeic acid	0.23 ± 0.02
6	Syringic acid	0.08 ± 0.01
7	Epicatechin	1.22 ± 0.02
8	Rutin	3.56 ± 0.26
9	Hyperoside	5.95 ± 0.19
10	Benzoic acid	0.81 ± 0.03
11	Quercitrin	7.81 ± 0.01
12	Quercetin	0.15 ± 0.01
13	Kaempferol	1.49 ± 0.41
14	Resveratrol	0.87 ± 0.02

### Effect of mulberry leaf polyphenols on body weight in obese mice

**FIGURE 1:**
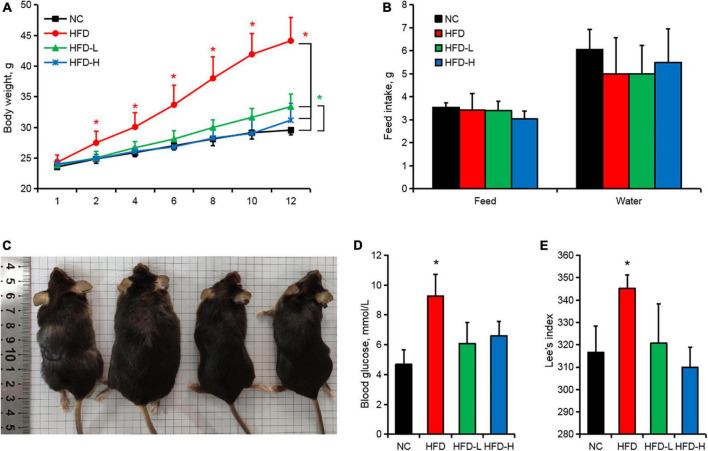
Mulberry leaf polyphenols (MLP) alleviated the weight gain induced by the high-fat diet (HFD) in mice. **(A)** Mouse body weight change curve. **(B)** The average daily food intake and water intake of the mice throughout the whole period. **(C)** Images of mouse body shapes after 12 weeks of different treatments. **(D)** Fasting blood glucose of mice fasted for 12 h. **(E)** The Lee’s index of mice after 12 weeks of different treatments. *P ≤ 0.05, *n* = 10.

The weights of the HFD-induced mice were significantly higher than those of the control group from the second week (Figures 1A,C). The gavage of MLP did not affect the feeding and drinking habit of mice (Figure 1B). However, the gavage of MLP significantly reduced the weight gain (Figure 1A), fasting blood glucose (Figure 1D), and Lee’s index (Figure 1E) of the HFD-induced mice.

### Effects of mulberry leaf polyphenols on organ development in high-fat diet-induced obese mice

**FIGURE 2:**
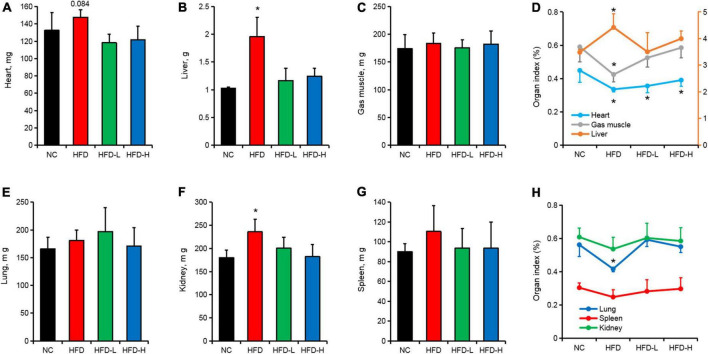
Effect of mulberry leaf polyphenols (MLP) on the organs of obese mice. **(A–C)** The weight of the heart **(A)**, liver **(B)**, and gastrocnemius **(C)** of mice after 12 weeks of MLP and high-fat diet (HFD) treatments. **(D)** Mice heart, liver, and gastrocnemius organ index after 12 weeks of MLP and HFD treatments. **(E–G)** The weight of the lungs **(E)**, kidney **(F)**, and spleen **(G)** of mice after 12 weeks of MLP and HFD treatments. **(H)** Mice lung, kidney, and spleen organ index after 12 weeks of MLP and HFD treatments. *P ≤ 0.05, *n* = 6.

In addition to inducing obesity in mice, the HFD significantly increased the weight of the liver (Figure 2B) and kidneys (Figure 2F) in mice, but it had no significant effect on the weight of the heart (Figure 2A), gastrocnemius muscle (Gas muscle, Figure 2C), lungs (Figure 2E), and spleen (Figure 2G). Additionally, the HFD significantly increased the liver index of mice and significantly reduced the heart index, gastrocnemius index, and lung index (Figures 2D,H).

### Effect of mulberry leaf polyphenols on the adipose tissue of high-fat diet-induced obese mice

**FIGURE 3:**
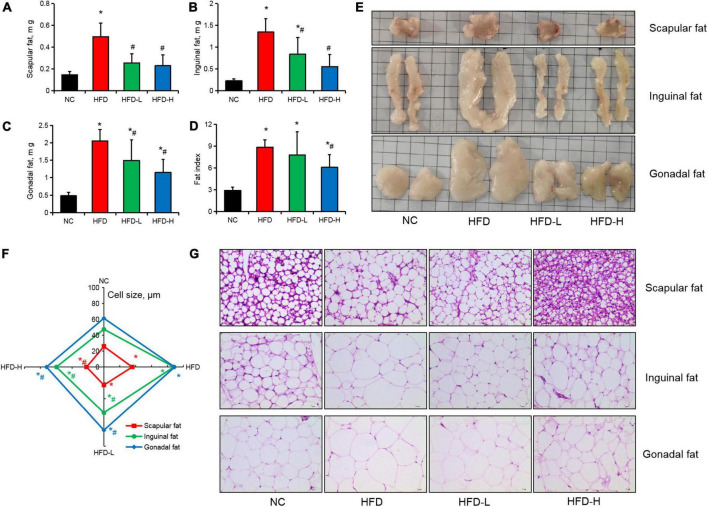
Mulberry leaf polyphenols (MLP) reduced fat gain in obese mice. **(A–C)** The scapular **(A)**, inguinal **(B)**, and gonadal **(C)** fat weights of mice after 12 weeks of MLP and high-fat diet (HFD) treatment. **(D)** The fat index after 12 weeks of MLP and HFD treatment (contains scapular, inguinal, and gonadal fats). **(E)** Images of adipose tissue in different parts of a mouse. **(F)** The size distribution of fat cells in different parts of the adipose tissues in mice. **(G)** H&E staining of scapular, inguinal, and gonadal fats. *P ≤ 0.05, compared with the NC group; #*P* ≤ 0.05, compared with the HFD group, *n* = 6.

High-fat diet significantly increased the weight of scapular, inguinal, and gonadal fats in the mice, while low-dose and high-dose MLP significantly inhibited fat gain in different parts of the mice (Figures 3A–C,E). For the fatty organ index, only a high concentration of MLP significantly alleviated the increase in fatty organ index induced by the HFD (Figure 3D). Moreover, the H&E staining of adipose tissue and fat cell size determination results showed that a high concentration of MLP significantly inhibited the increase in fat cell volume in different parts of the mice induced by the HFD. In contrast, low-concentration MLP significantly inhibited the inguinal and gonadal fats (Figures 3F,G).

### Effect of mulberry leaf polyphenols on the fatty acid composition of adipose tissues in mice

**FIGURE 4:**
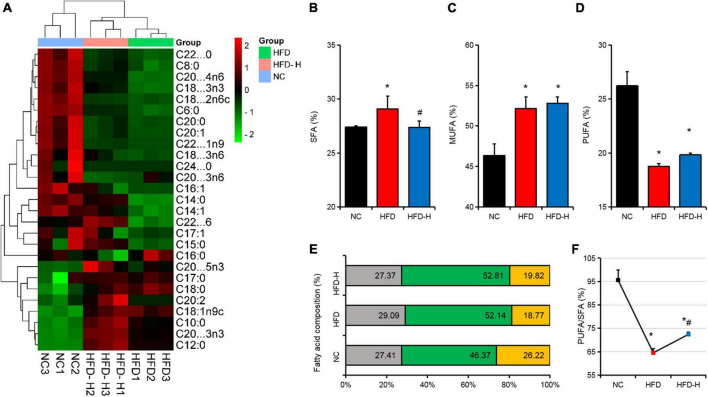
Mulberry leaf polyphenols (MLP) changed the fatty acid composition of the adipose tissues in obese mice. **(A)** The heat map shows the fatty acid composition of the adipose tissues in mice with different treatments. **(B–D)** The composition of saturated fatty acids (SFA, **B**), monounsaturated fatty acids (MUFA, **C**), and polyunsaturated fatty acids (PUFA, **D**) in mice adipose tissues. **(E)** SFA, MUFA, and PUFA composition distribution stacking diagram of mouse adipose tissues. **(F)** Mice adipose tissue PUFA/SFA ratio. *P ≤ 0.05, compared with the NC group; #*P* ≤ 0.05, compared with the high-fat diet (HFD) group. *n* = 3.

The low concentration of MLP had no significant effect on the fat organ index and scapular fossa fat cell morphology (Figures 3D,F). Therefore, we analyzed the fatty acids in the adipose tissues of mice in the NC, HFD, and HFD-H groups and found that the fatty acid composition of the three groups was clustered separately (Figure 4A). Further analysis indicated that the HFD significantly promoted SFA and MFA content, but significantly reduced the PUFA content and PUFA/SFA ratio (Figures 4B–F). Compared with the HFD group, a high concentration of MLP significantly reduced the SFA content and increased the PUFA/SFA ratio (Figures 4B,F).

### The effect of mulberry leaf polyphenols on the expression of characteristic genes in the adipose tissues of obese mice

**FIGURE 5:**
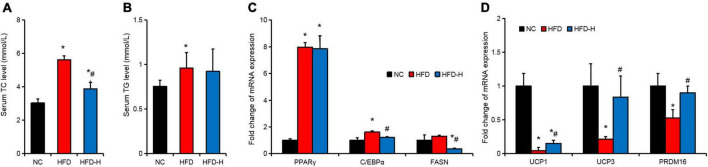
Mulberry leaf polyphenols (MLP) promoted the expression of thermogenesis genes in the adipose tissues of obese mice. **(A,B)** Mice serum total cholesterol (TC, **A**) and triglyceride (TG, **B**) content of mice with different treatments. **(C)** Expression of genes related to fat deposition in the adipose tissues of mice with different treatments. **(D)** The expression of browning-related genes in the adipose tissues of mice with different treatments. *P ≤ 0.05, compared with the NC group; #*P* ≤ 0.05, compared with the high-fat diet (HFD) group. *n* = 3.

The HFD significantly promoted the serum TC and TG levels. The MLP significantly inhibited the serum TC level induced by the HFD but did not significantly inhibit the serum TG level (Figures 5A,B). The expression of genes related to fat deposition and adipose tissue browning in the adipose tissues was detected using RT-qPCR. The results indicate that HFD significantly promoted the expression of PPARγ and C/EBPα, but significantly inhibited the expression of browning-related genes, such as UCP1, UCP3, and PRDM16 (Figures 5C,D). Compared with the HFD group, high concentrations of MLP significantly inhibited the expression of FASN and significantly promoted the expression of UCP1, UCP3, and PRDM16 (Figures 5C,D).

## Discussion

Obesity has become a worldwide public health concern. The imbalance in energy intake and output is the primary cause of obesity, leading to excessive body fat accumulation (22). Moreover, factors, such as genetics, physiology, nutrition, and environment, may also contribute to the occurrence of obesity (23). Obesity often leads to various chronic diseases, such as hypertension, dyslipidemia, type 2 diabetes, cardiovascular disease, insomnia, and apnea (7). According to reports, the global rate of overweight and obesity has increased to 40%, affecting more than 2 billion people (24). Therefore, obesity intervention has become a key concern of the whole society. The increasing changes in lifestyle, increased work pressure, and the safety and tolerance of drug treatments have influenced increasingly more people to opt for functional foods with anti-obesity effects (25). Phytochemical ingredients are recognized as safe because they have no side effects within a certain concentration range, and hence, are favored by most researchers and consumers. ML Pare one of the natural products with potential effects of lipid-lowering, antioxidant, and weight loss (26). In this study, the effects of MLP on fat deposition in obese mice were assessed.

In recent years, many polyphenols have been extracted from various plants, such as apples, tea, grapes, and blueberries (27–29). Polyphenols are characterized by antioxidant, antibacterial, and antiviral activities. Regarding the treatment of metabolic diseases, MLP have the characteristic effects of lowering blood sugar, lowering blood pressure, and preventing cardiovascular diseases (30). Our study results proved that both low and high concentrations of MLP could significantly reduce the level of fasting blood glucose in HFD-induced obese mice. Additionally, they could reduce serum triglycerides and total cholesterol levels in obese mice.

Stimulating the body’s heat production and accelerating energy consumption are effective approaches to losing weight (31). Studies have reported that the anti-obesity effect of polyphenols might be attributed to the heat production stimulation in the body (25). Previous studies have found that green tea extract could increase and prolong sympathetic nerve stimulation and heat production, and effectively reduce body weight and fat deposition in the body organs (32). The results of the present study indicated that MLP could significantly upregulate the expression of brown fat thermogenesis-related genes, such as UCP1, UCP3, and PRDM16 (33, 34).

The results of this study revealed that MLP could change the fatty acid composition of adipose tissues in obese mice. Obesity leads to increased fat deposition and affects the composition of fatty acids in tissues (35). Studies have reported that in children and adolescents, 16:0 and 18:0 are positively correlated with obesity, while 20:4n-6 and 22:6n-3 are negatively correlated with obesity (36). In this study, the MLP downregulated 16:0 and 18:0 in obese mice and upregulated the 20:4n-6 and 22:6n-3 content. The fatty acid composition of food also affects the occurrence of obesity, while a higher SFA is not conducive to body health (37–39). This study found that MLP could downregulate the SFA content of obese mice. The PUFA/SFA ratio is also closely related to health, with an approximate ratio of 1 (40). The current results show that the PUFA/SFA ratio of obese mice was only 65%, and MLP significantly increased the PUFA/SFA ratio of obese mice. Therefore, using MLP to feed domestic animals might improve the quality of livestock products by changing their fatty acid composition (41).

## Conclusion

The results of this study show that MLP could alleviate HFD-induced obesity in mice fat deposition reduction. Moreover, MLP could reduce the expression of fat synthesis-related genes and increase the expression of browning-related genes in the adipose tissues of HFD-induced obese mice. In addition, this study found that MLP may also affect the health of obese mice by changing the fatty acid composition of adipose tissue. Overall, MLP could be potential weight-loss drugs. This study provides a reference for developing animal feed additives using MLP.

## Data availability statement

The original contributions presented in this study are included in the article/Supplementary material, further inquiries can be directed to the corresponding author.

## Ethics statement

This animal study was reviewed and approved by the Animal Care and Ethics Committee of Chongqing Academy of Animal Sciences.

## Author contributions

RL: conceptualization, methodology, software, investigation, formal analysis, funding acquisition, and writing—original draft and editing. QZ: software, data curation, detection, methodology, and writing—review and editing. XW: data curation, methodology, software, and validation. HW: conceptualization, funding acquisition, resources, supervision, and writing—review and editing. All authors contributed to the article and approved the submitted version.

## Abbreviations

MLP: mulberry leaf polyphenols
SFA: saturated fatty acids
MUFA: monounsaturated fatty acids
PUFA: polyunsaturated fatty acids
PPARγ: peroxisome proliferative activated receptor, gamma
C/EBPα: CCAAT/enhancer-binding protein alpha
FASN: fatty acid synthase
UCP1: uncoupling protein 1
UCP3: uncoupling protein 3
PRDM 16: PR domain-containing 16.
