# Supplementary material for: Mulberry leaf polyphenols alleviated high-fat diet-induced obesity in mice
Source: Front Nutr. 2022 Sep 15;9:979058. doi: 10.3389/fnut.2022.979058 (PMC9521161; doi:10.3389/fnut.2022.979058)
Supplement: Supplementary file 1 [file Table_1.pdf]

Table Supplementary

Table S1. The primer sequences

Gene	Sequence
m-PPARγ:F	GTGCCAGTTTCGATCCGTAGA
m-PPARγ:R	GGCCAGCATCGTGTAGATGA
m-C/EBPα:F	CAAGAACAGCAACGAGTACCG
m-C/EBPα:R	GTCACTGGTCAACTCCAGCAC
m-FASN-F	CCACTGCTTACTACTCGTTA
m-FASN-R	AGGTATGCTCGCTTCTCT
m-UCP1-F	AGGCTTCCAGTACCATTAGGT
m-UCP1-R	CTGAGTGAGGCAAAGCTGATTT
m-UCP3-F	CTGCACCGCCAGATGAGTTT
m-UCP3-R	ATCATGGCTTGAAATCGGACC
m-PRDM16:F	CCACCAGCGAGGACTTCAC
m-PRDM16:R	GGAGGACTCTCGTAGCTCGAA
m-ACTB:F	GCTGTATTCCCCTCCATCGT
m-ACTB:R	CTTCTCCATGTCGTCCCAGT
